# Nomogram Predicting the Benefits of Adding Concurrent Chemotherapy to Intensity-Modulated Radiotherapy After Induction Chemotherapy in Stages II–IVb Nasopharyngeal Carcinoma

**DOI:** 10.3389/fonc.2020.539321

**Published:** 2020-11-09

**Authors:** Sai-Lan Liu, Xue-Song Sun, Zi-Jian Lu, Qiu-Yan Chen, Huan-Xin Lin, Lin-Quan Tang, Jin-Xin Bei, Ling Guo, Hai-Qiang Mai

**Affiliations:** ^1^ Sun Yat-sen University Cancer Center, State Key Laboratory of Oncology in South China, Collaborative Innovation Center for Cancer Medicine, Guangdong Key Laboratory of Nasopharyngeal Carcinoma Diagnosis and Therapy, Guangzhou, China; ^2^ Department of Nasopharyngeal Carcinoma, Sun Yat-sen University Cancer Center, Guangzhou, China; ^3^ Department of Radiation Oncology, Sun Yat-sen University Cancer Center, Guangzhou, China

**Keywords:** nasopharyngeal carcinoma (NPC), induction chemotherapy (IC), concurrent chemoradiotherapy, radiotherapy, nomogram

## Abstract

**Background:**

To compare the efficacy of induction chemotherapy plus concurrent chemoradiotherapy (IC+CCRT) versus induction chemotherapy plus radiotherapy (IC+RT) in patients with locoregionally advanced nasopharyngeal carcinoma (NPC).

**Patients and Methods:**

One thousand three hundred twenty four patients with newly-diagnosed NPC treated with IC+CCRT or IC+RT were enrolled. Progression-free survival (PFS), distant metastasis-free survival (DMFS), overall survival (OS), locoregional relapse-free survival (LRFS), and acute toxicities during radiotherapy were compared using propensity score matching (PSM). A nomogram was developed to predict the 3- and 5-year PFS with or without concurrent chemotherapy (CC).

**Results:**

PSM assigned 387 patients to the IC+CCRT group and IC+RT group, respectively. After 3 years, no significant difference in PFS (84.7 vs. 87.5%, P = 0.080), OS (95.5 vs. 97.6%, P = 0.123), DMFS (89.7 vs. 92.8%, P = 0.134), or LRFS (94.0 vs. 94.1%, P = 0.557) was noted between the groups. Subgroup analysis indicated comparable survival outcomes in low-risk NPC patients (II–III with EBV DNA <4,000 copies/ml) between the groups, although IC+RT alone was associated with fewer acute toxicities. However, IC+CCRT was associated with significantly higher 3−year PFS, OS, DMFS, and LRFS rates, relative to IC+RT alone, in high-risk NPC patients (IVa-b or EBV DNA ≥4,000 copies/ml). Multivariate analysis showed that T category, N category, EBV DNA level, and treatment group were predictive of PFS, and were hence incorporated into the nomogram. The nomogram predicted that the magnitude of benefit from CC could vary significantly.

**Conclusions:**

IC+RT had similar efficacy as IC+CCRT in low-risk NPC patients, but was associated with fewer acute toxicities. However, in high-risk patients, IC+CCRT was superior to IC+RT.

## Introduction

Nasopharyngeal carcinoma (NPC), a malignant head and neck cancer arising from nasopharynx epithelia, is ethnically specific and endemic to southern China, Southeast Asia, North Africa, the Middle East, and Alaska (1–3). Due to the specific anatomical location and sensitive biological characteristics, radiotherapy (RT) is the backbone of NPC treatment (4). Patients in early tumor stage can be cured by RT alone. However, more than 70% of newly diagnosed NPC patients are locoregionally advanced diseases with poor prognosis (5). RT plus concurrent chemotherapy (CC) with or without adjuvant chemotherapy (AC) has been established as the standard treatment schedule for these patients (6–11). Recently, the benefits of adding induction chemotherapy (IC) to the previously concurrent chemoradiotherapy (CCRT) regimen have attracted an increasing amount of attention (12). According to randomized phase III clinical trials, the application of IC prior to CCRT could improve the prognosis of locoregionally advanced NPC (13, 14).

However, due to the toxicity of CC, a high incidence of grade 3–4 adverse events is often observed, which reduces patients’ compliance and lead to the failure of completing subsequent radiotherapy plan (15). Moreover, Wang et al. (4). demonstrated that IC+RT and IC+CCRT had similar survival outcomes in stage II–IVb patients, while IC+RT had lower treatment-related grade 3/4 acute hematological toxicity than IC+CCRT group during radiation. The same conclusion was mentioned in previous studies (16–18). Though previous studies discussed and compared the toxicity, efficacy, and benefits of IC+RT and IC+CCRT. However, the addition of CC to RT after IC remains controversial. To our knowledge, there is no such study investigating which kind of patients is suitable to accept CCRT and which kind of patients is unnecessary to add CC during RT after IC. Based on the abovementioned findings, we believe that there may be certain stage II–IVb patients with a low risk of treatment failure. For these patients, intensity-modulated radiotherapy (IMRT) alone was sufficient to achieve an equivalent curative effect, relative to CCRT after IC, although the addition of CC was necessary for other high-risk patients. As present, no study has measured the efficacy of these 2 strategies among patients with different risk factors.

Nowadays, risk-classification tools have been developing including machine learning, artificial neural network, and nomogram and etc. (19–22). These techniques are now more and more used for NPC patients in helping clinical decision-making. A nomogram is a prediction tool widely used for risk quantification. This tool has been proven to generate more precise predictions of prognosis, such as overall survival (OS) and progression-free survival (PFS), in several types of cancers, including NPC (23–25). Since previous studies did not stratify the patients suitable for CC following IC, a nomogram combining potential risk factors may provide more information. In the present study, we aimed to predict the prognosis combining EBV DNA and tumor stage using a nomogram and to estimate the benefit and of CC following IC based on data from a cohort of newly diagnosed stage II–IV NPC patients, which could be useful for guiding individualized treatment.

## Patients and Methods

Patients with histologically-diagnosed NPC who were treated at Sun Yat-Sen Cancer Center from 2009 to 2017 were retrospectively reviewed. The eligibility criteria included the following: (1) biopsy proven World Health Organization (WHO) histopathologic types II or III NPC; (2) 18 years of age or older; (3) stage II–IVb according to the 7th edition of America Joint Committee on Cancer staging system (26); (4) received IC plus IMRT with or without CC; (5) complete data of pre-treatment plasma Epstein–Barr virus (EBV) DNA level; and (6) adequate hematologic, liver, and renal function. The exclusion criteria were as follows: stage I NPC, wherein distant metastasis had developed at diagnosis; incomplete treatment information; dysfunction of the liver or kidney; presence of a second primary tumor or history of malignant tumors; receiving adjuvant chemotherapy; and receiving targeted drugs.

### Chemotherapy and Radiation Therapy

All patients were treated with IC followed by RT (n = 387) or CCRT (n = 937). The IC regimen consisted of TPF [consisting of docetaxel (60 mg/m^2^ on day 1), paclitaxel (135 mg/m^2^ on day 1), paclitaxel liposome (135 mg/m^2^ on day 1); cisplatin (60 mg/m^2^ on day 1); and 5-fluorouracil (500–800 mg/m^2^, 120 h of continuous intravenous infusion)] or PF [consisting of cisplatin (80–100 mg/m^2^ on day 1) and 5-fluorouracil (800–1,000 mg/m^2^, 120 h of continuous intravenous infusion)] and TP [consisting of docetaxel (75 mg/m^2^ on day 1), paclitaxel (150–180 mg/m^2^ on day 1), or paclitaxel liposome (150–180 mg/m^2^ on day 1) and cisplatin (20–25 mg/m^2^ on day 1–3)] for 2–3 cycles. RT, conducted as IMRT, was administered 2–3 weeks after IC at the nasopharyngeal and cervical lymph nodes, using a 6-MV X-ray with a total dose of 68–72 Gy at the primary tumor and 64–70 Gy at the cervical lymph nodes in 30–33 fractions. Moreover, patients in the CCRT group received cisplatin-based chemotherapy during RT (80–100 mg/m^2^, every 3 weeks or 30–40 mg/m^2^ weekly).

### Clinical Outcome and Follow-Up

Blood cell counts and serum chemistry profiles were examined during the treatment process to monitor the toxic reactions. Therapy-related toxicities were evaluated according to Common Terminology Criteria for Adverse Events 4.0. Follow-up visits were initiated 3 months after the last day of therapy, and this frequency was maintained for 3 years, after which it was altered to 6-month intervals until death. Each follow-up included at least a complete physical examination, nasopharyngeal endoscopy, head, and neck magnetic resonance imaging (MRI), chest radiography/chest computed tomography (CT), abdominal sonography/abdominal CT, and EBV-DNA test. Additional examinations, such as bone scan and positron emission tomography/computed tomography (PET/CT), were recommended when necessary.

The primary endpoint of our study was PFS (the date of treatment initiation to the first failure at any site or death of any cause or patient censorship at the date of the last follow-up). OS (duration from treatment to death of any cause or the date of the last follow-up), locoregional recurrence-free survival (LRFS; time from treatment initiation until recurrence in the nasopharyngeal or neck area), distant metastasis-free survival (DMFS; time from treatment initiation until the detection of distant metastasis), and toxicity were the secondary endpoints.

### Statistical Analysis

We used propensity score matching (PSM) to compare survival outcomes and toxicities between the 2 groups. Kaplan-Meier survival curve analysis was used to assess the time-to-event endpoints and the log-rank test was used to compare the differences in the PSM cohort. Multivariable analyses were performed using Cox proportional hazards model to test the independent statistical significance of the prognosis factors. A nomogram based on the Cox regression model was used to predict the 3- and 5-year PFS following the 2 different regimens. The C-index was calculated to measure the predictive accuracy and discriminative ability of this nomogram. Calibration curves were generated to estimate the performance of the nomogram along with bootstrap validation. A two-sided P value of <0.05 was considered significant. All these computations were performed with SPSS 24.0 (IBM Corporation, USA) or R 3.5.3 (R project, http://www.R-project.org/).

## Results

From 2009 to 2017, 1,324 stage II–IVb NPC patients were enrolled in the current study. The clinical characteristics grouped by treatment method are listed in **Table 1**. In the observational cohort, 387 patients received IC+RT alone and 937 patients received IC+CCRT. Patients presenting with advanced disease (T4, N3, and stage IV disease) were significantly more likely to undergo IC+CCRT (P < 0.001). To eliminate these potential confounding factors, we established the well-balanced cohort *via* PSM in the ratio of 1:1. Overall, 774 patients were identified for the matched analysis. There was no significant difference in the clinical characteristics between the 2 treatment methods in the PSM cohort.

**Table 1:** Differences in patient characteristics between the IC+RT and IC+CCRT groups in the observational and propensity-matched datasets.

Characteristic	Observational dataset (n = 1,324)		P	PSM dataset (n = 774)		P
	IC+RT	IC+CCRT		IC+RT	IC+CCRT	
**Total**	387	937		387	387	
**Age, years**			0.025^a^			0.062^a^
Median (range)	45(18–73)	43(20–74)		45(18–73)	43(20–70)	
<45	184(47.5)	509(54.3)		184(47.5)	210(54.3)	
≥45	203(52.5)	428(45.7)		203 (52.5)	177(45.7)	
**Sex**			0.114^a^			0.810^a^
Female	109(28.2)	225(24.0)		109(28.2)	106(27.4)	
Male	278(71.8)	712(76.0)		278(71.8)	281(72.6)	
**Pathologic (WHO) type**			0.370^b^			0.194^b^
I	0(0.0)	3(0.3)		0(0.0)	3(0.8)	
II	4(1.0)	5(0.5)		4(1.0)	2(0.5)	
III	383(99.0)	929(99.1)		383(99.0)	382(98.7)	
T stage*			<0.001^a^			0.879^a^
T1	10(2.6)	19(2.0)		10(2.6)	12(3.1)	
T2	59(15.2)	100(10.7)		59(15.2)	52(13.4)	
T3	260(67.2)	446(47.6)		260(67.2)	263(68.0)	
T4	58(15.0)	372(39.7)		58(15.0)	60(15.5)	
N stage*			<0.001^a^			0.147^a^
N0	22(5.7)	22(2.3)		22(5.7)	13(3.4)	
N1	150(38.8)	244(26.0)		150(38.8)	135(34.9)	
N2	184(47.5)	446(47.6)		184(47.5)	212(54.8)	
N3	31(8.0)	225(24.0)		31(8.0)	27(7.0)	
Clinical stage*			<0.001^a^			0.364^a^
II	29(7.5)	18(1.9)		29(7.5)	18(4.7)	
III	276(71.3)	389(41.5)		276(71.3)	289(74.7)	
IVa	51(13.2)	305(32.6)		51(13.2)	53(13.7)	
IVb	31(8.0)	225(24.0)		31(8.0)	27(7.0)	
**EBV DNA (copies/ml)**			<0.001^a^			0.474^a^
<4,000	281(72.6)	461(49.2)		281(72.6)	272(70.3)	
≥4,000	106(27.4)	476(50.8)		106(27.4)	115(29.7)	

IC, induction chemotherapy; RT, radiotherapy; CCRT, concurrent chemoradiotherapy; WHO, World Health Organization; EBV, Epstein–Barr virus.

^a^P value calculated using the chi-square test. ^b^P value calculated using Fisher’s exact test.

^*^According to the 7th edition of the UICC/AJCC staging system.

### Risk Stratification

Patients with different TNM stages and pre-EBV DNA levels would exhibit different rates of treatment failure. Therefore, we divided patients into 2 subgroups based on the TNM stage or EBV DNA level, and compared their survival outcomes in terms of PFS. The cut-off value for the EBV DNA level (4,000 copies/ml) was determined in a previous study and through clinical practice (27–29). In an unadjusted analysis, patients with stage IV disease and higher EBV DNA level (≥ 4,000 copies/ml) were found to be more likely to have lower PFS (P < 0.001). Kaplan–Meier survival curves are shown in **Figures S1A, B**. Based on these 2 prognostic factors, we further subdivided all patients into 3 subgroups: group A, stage II–III cases with EBV DNA <4,000 copies; group B, stage II–III cases with EBV DNA ≥4,000 copies or stage IV cases with EBV DNA <4,000 copies; and group C, stage IV cases with EBV DNA ≥4,000 copies. As the prognosis of patients in group A was much better than that of patients in groups B and C, we combined groups B and C into the high-risk subgroup and group A served as the low risk subgroup. The Kaplan-Meier survival curves are shown in **Figures S1C, D**.

### Efficacy of CC in Different Risk Subgroups

**Figure 1:**
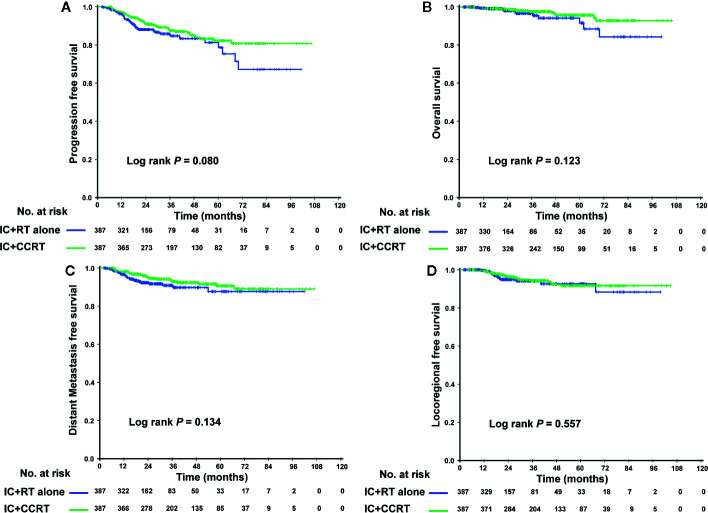
Kaplan–Meier curves of the PSM cohort who received the IC+RT regimen or the IC+CCRT regimen in terms of PFS **(A)**, OS **(B)**, DMFS **(C)**, and LRFS **(D)**. PSM, propensity score matching; IC, induction chemotherapy; RT, radiotherapy; CCRT, concurrent chemoradiotherapy; PFS, progression-free survival; OS, overall survival; DMFS, distant metastasis-free survival; LRFS, locoregional relapse-free survival.

**Figure 2:**
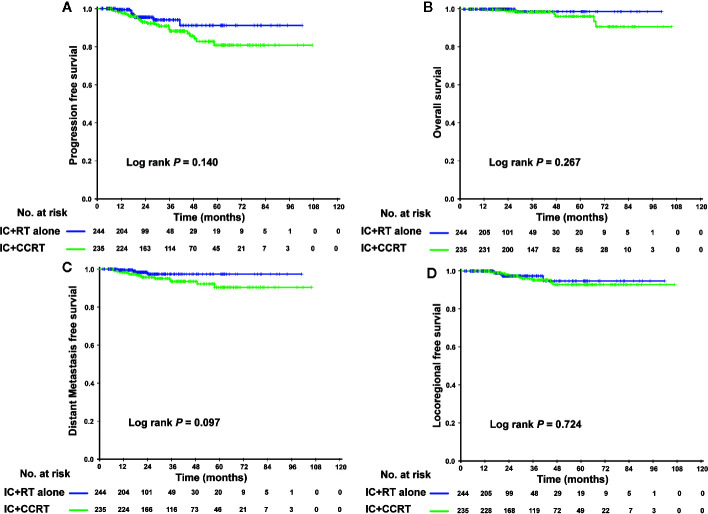
Kaplan–Meier curves of the 479 low-risk NPC patients (II–III stage and EBV DNA <4,000 copies/ml) who received the IC+RT regimen or IC+CCRT regimen in terms of PFS **(A)**, OS **(B)**, DMFS **(C)**, and LRFS **(D)**. NPC, nasopharyngeal carcinoma; EBV DNA, Epstein–Barr virus DNA; IC, induction chemotherapy; RT, radiotherapy; CCRT, concurrent chemoradiotherapy; PFS, progression-free survival; OS, overall survival; DMFS, distant metastasis-free survival; LRFS, locoregional relapse-free survival.

**Figure 3:**
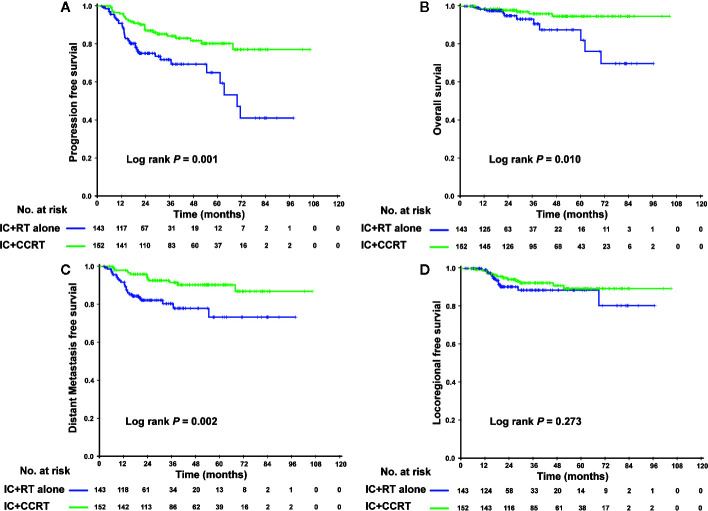
Kaplan–Meier curves of the 295 high-risk NPC patients (IVa–b stage or EBV DNA ≥4,000 copies/ml) who received the IC+RT regimen or IC+CCRT regimen in terms of PFS **(A)**, OS **(B)**, DMFS **(C)**, and LRFS **(D)**. NPC, nasopharyngeal carcinoma; EBV DNA, Epstein–Barr virus DNA; IC, induction chemotherapy; RT, radiotherapy; CCRT, concurrent chemoradiotherapy; PFS, progression-free survival; OS, overall survival; DMFS, distant metastasis-free survival; LRFS, locoregional relapse-free survival.

In the PSM cohort, we investigated whether patients could benefit from the application of CC after IC. However, there were no significant differences in the risk of death, disease progression, distant metastasis, and loco-regional relapse. The differences in efficacy between the 2 groups are shown in **Figure 1**. Patients who underwent IC+RT alone (relative to those who underwent IC+CCRT) had 3-year PFS, OS, DMFS, and LRFS rates of 84.7 vs. 87.5% (P = 0.080), 95.5 vs. 97.6% (P = 0.123), 89.7 vs. 92.8% (P = 0.134), and 94.0 vs. 94.1% (P = 0.557), respectively. Given that patients with different risk stratification had different benefits from CC, we further explored the efficacy of CC in different risk subgroups. Interestingly, CC showed different curative effects between the 2 risk subgroups. Among the low-risk patients (pre-EBV DNA <4,000 copies and stage III), the 3-year PFS, OS, LRFS, and DMFS rates were similar between the IC+RT alone group and IC+CCRT group (**Figure 2**). However, in the high-risk patients (pre-EBV DNA ≥4,000 copies or stage IV), the 3-year PFS, OS, and DMFS rates were higher in the IC+CCRT group than in the IC+RT alone group (3-year PFS rate: 84.5 vs. 70.0% P = 0.001; 3-year OS rate: 96.0 vs. 90.7% P = 0.010; 3-year DMFS rate: 91.6 vs. 78.3% P = 0.002; 3-year LRFS rate: 92.4 vs. 88.6% P = 0.273). The Kaplan–Meier curves are shown in **Figure 3**.

### Establishing the Nomogram

**Figure 4:**
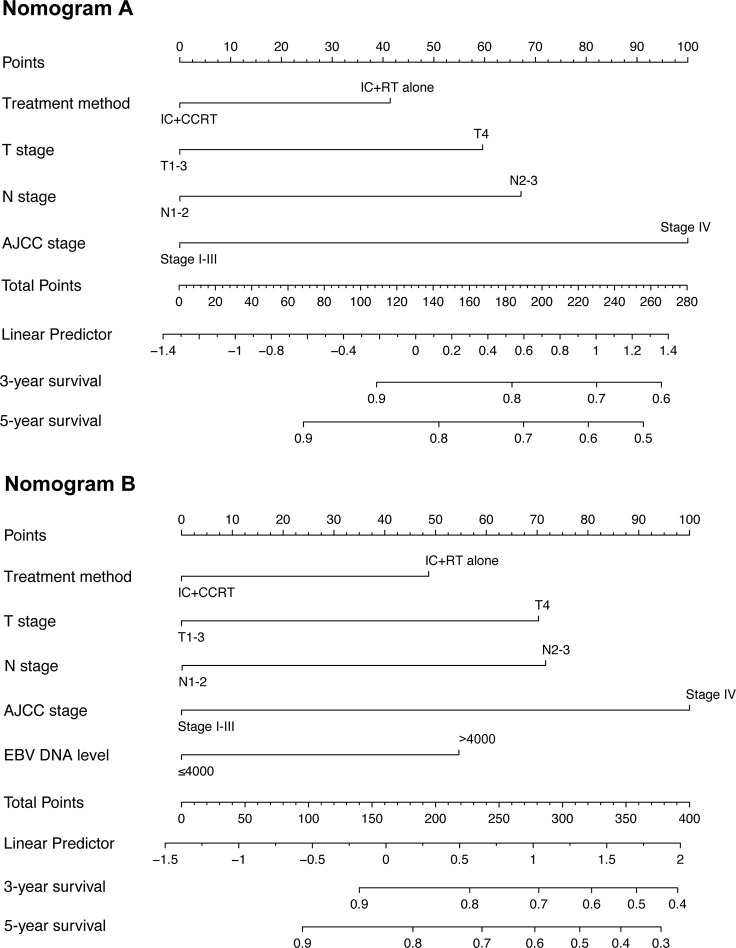
Nomogram for 3- and 5-year progression-free survival (PFS) in patients with nasopharyngeal carcinoma. The nomogram allows the user to obtain the probability of 3- and 5-year PFS based on a patient’s combination of covariates. For example, the patient’s T stage is identified, and a line is drawn straight upward to the “Points” axis to determine the score associated with that T stage. The process is repeated for each variable, the scores are summed for each covariate, and the sum is determined on the “Total Points” axis. A line is drawn straight down to determine the likelihood of 3- or 5-year PFS. EBV DNA, Epstein–Barr virus DNA; IC, induction chemotherapy; RT, radiotherapy; CCRT, concurrent chemoradiotherapy; PFS, progression-free survival.

**Figure 5:**
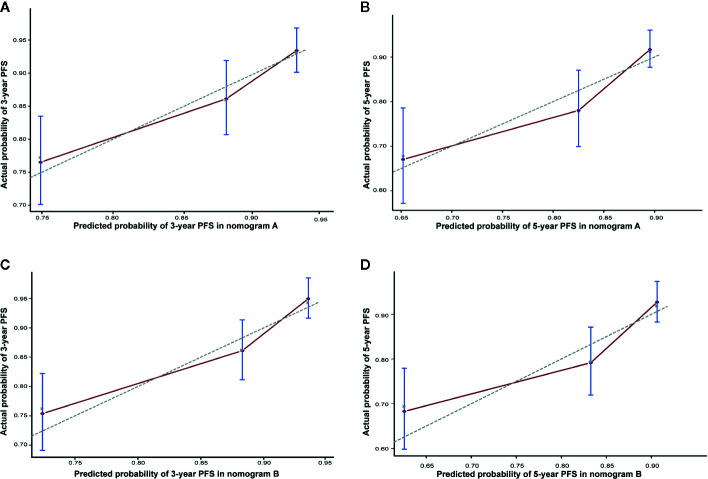
Calibration curve of nomogram A for predicting progression-free survival (PFS) at **(A)** 3 years and **(B)** 5 years in the matched cohort. Calibration curve of nomogram B for predicting progression-free survival (PFS) at **(C)** 3 years and **(D)** 5 years in the matched cohort. The actual PFS is plotted on the y-axis; the nomogram predicts the probability of PFS, which is plotted on the x-axis.

To predict the prognosis of each patient, we established a nomogram based on the PFS using a Cox regression model. In the PSM cohort, all the potential prognostic factors were included in the multivariate analysis. Finally, we identified T stage [T4 vs. T1–2: hazard ratio (HR) = 3.784, 95% confidence interval (CI) = 1.822–7.861, P < 0.001; T3 vs. T1–2: HR = 1.761, 95% CI = 0.896–3.464, P = 0.101], N stage (N3 vs. N0-1: HR = 2.962, 95% CI = 1.538–5.705, P = 0.001; N2 vs. N0-1: HR = 1.893, 95% CI = 1.180–3.036, P = 0.008), EBV DNA level (≥4,000 vs. <4,000 copies/ml: HR = 1.667, 95% CI = 1.091–2.546, P = 0.018), and treatment method (IC+CCRT vs. IC+RT alone: HR = 0.658, 95% CI = 0.437–0.990, P = 0.045) as the independent prognostic factors (**Table 2**), which were then used to establish the nomogram model. The 3-year PFS and 5-year PFS could be predicted easily by adding up the score of each prognostic factor and locating the total score on the point scale (**Figure 4**). Moreover, the survival benefit of the addition of CC to IC could be estimated for individual patients with different risk factors. The nomogram model showed good accuracy for predicting PFS with a C index of 0.703 (95% CI, 0.644–0.762) using the bootstrap validation method. The calibration curves also presented acceptable agreement between the nomogram-predicted value and actual value for 3- and 5-year PFS (**Figure 5**).

**Table 2:** Summary of multivariate analyses of prognostic factors for 3-year progression-free survival in 774 matched NPC cases.

	Hazard ratio (95% CI)	P value
**Progression-free survival**		
Age (y) (≥45 vs. <45)	1.159(0.769–1.747)	0.481
Gender (F vs. M)	0.960(0.614–1.502)	0.858
T category; T3 vs. T1-2	1.761(0.896–3.464)	0. 101
T category; T4 vs. T1-2	3.784(1.822–7.861)	<0.001
N category; N2 vs. N0-1	1.893(1.180–3.036)	0.008
N category; N3 vs. N0-1	2.962(1.538–5.705)	0.001
EBV DNA	1.667(1.091–2.546)	0.018
Regimen; IC+CCRT vs. IC+RT	0.658(0.437–0.990)	0.045
**Overall survival**		
Age (y) (≥45 vs. <45)	1.365(0.596–3.125)	0.462
Gender (M vs. F)	1.267(0.494–3.252)	0.622
T category; T3 vs. T1-2	1.806(0.397–8.209)	0. 444
T category; T4 vs. T1-2	8.378(1.822–38.522)	0.006
N category; N2 vs. N0-1	0.864(0.369–2.024)	0.736
N category; N3 vs. N0-1	1.749(0.483–6.330)	0.394
EBV DNA	0.735(0.312–1.734)	0.482
Regimen; IC+CCRT vs. IC+RT	0.638(0.285–1.430)	0.275
**Distant metastasis-free survival**		
Age (y) (≥45 vs. <45)	1.587(0.924–2.726)	0.094
Gender (M vs. F)	0.803(0.454–1.421)	0.451
T category; T3 vs. T1–2	1.697(0.710–4.058)	0. 234
T category; T4 vs. T1–2	2.600(0.991–6.824)	0. 052
N category; N2 vs. N0–1	1.902(1.008–3.590)	0.047
N category; N3 vs. N0–1	3.145(1.352–7.316)	0.008
EBV DNA	2.113(1.209–3.695)	0.009
Regimen; IC+CCRT vs. IC+RT	0.639(0.375–1.090)	0.100
**Locoregional relapse-free survival**		
Age (y) (≥45 vs. <45)	0.883(0.461–1.690)	0.707
Gender (M vs. F)	1.223(0.589–2.540)	0.589
T category; T3 vs. T1–2	1.788(0.613–5.214)	0. 287
T category; T4 vs. T1–2	4.734(1.509–14.847)	0. 008
N category; N2 vs. N0–1	1.951(0.943–4.035)	0.072
N category; N3 vs. N0–1	2.758(0.935–8.137)	0.066
EBV DNA	1.173(0.599–2.297)	0.641
Regimen; IC+CCRT vs. IC+RT	0.776(0.407–1.477)	0.439

CI, confidence interval; EBV, Epstein–Barr virus; IC, induction chemotherapy; RT, radiotherapy; CCRT, concurrent chemoradiotherapy.

A Cox proportional hazards regression model was used to detect variables, one-by-one, without adjustment. All variables were transformed into categorical variables. HRs were calculated for age (≥45 vs. <45 years), sex (female vs. male), T stage (3 vs. 1–2; 4 vs. 1–2), N stage (2 vs. 0–1; 3 vs. 0–1), EBV DNA level (≥4,000 vs. <4,000 copies/ml), and regimen (IC+CCRT vs. IC+RT).

### Acute Toxicity

We evaluated acute toxicity between different treatment groups during the RT period in the PSM cohort. The rates of severe Grade 3–4 hematological toxicities were higher in the IC+CCRT group. A greater number of patients in the IC+CCRT group suffered from Grade 3–4 leukocytopenia (19.4 vs. 3.9%, P < 0.001), neutropenia (12.9 vs. 3.3%, P < 0.001), anemia (6.2 vs. 0.5%, P < 0.001), and thrombocytopenia (4.4 vs. 0.8%, P < 0.001), as compared to those in the IC+RT alone group. Moreover, a higher incidence of grade 1–4 nephrotoxicity was observed in the IC+CCRT group. No significant differences in terms of hepatoxicity were observed between the 2 treatment groups (**Table 3**).

**Table 3:** Grades 1–4 acute toxicities due to RT between the two groups.

Adverse event(toxicity grade)	IC+RT (n = 387)				IC+CCRT (n = 387)				P value for eventsgrade≥1	P value for events
	1(%)	2(%)	3(%)	4(%)	1(%)	2(%)	3(%)	4(%)		grade ≥3
Leukocytopenia	71(18.3)	66(17.1)	15(3.9)	0(0.0)	84(21.7)	181(46.8)	70(18.1)	5(1.3)	<0.001^a^	<0.001^a^
Neutropenia	38(9.8)	46(11.9)	9(2.3)	4(1.0)	118(30.5)	100(25.8)	46(11.9)	4(1.0)	<0.001^a^	<0.001^a^
Anemia	103(26.6)	21(5.4)	2(0.5)	0(0.0)	170(43.9)	126(32.6)	19(4.9)	5(1.3)	<0.001^a^	<0.001^b^
Thrombocytopenia	16(4.1)	6(1.6)	3(0.8)	0(0.0)	68(17.6)	30(7.8)	11(2.8)	6(1.6)	<0.001^a^	0.002^b^
AST increase	43(11.1)	3(0.8)	1(0.3)	0(0.0)	62(16.0)	3(0.8)	0(0.0)	0(0.0)	0.066^a^	1.000^b^
ALT increase	78(20.2)	13(3.4)	1(0.3)	0(0.0)	97(25.1)	9(2.3)	1(0.3)	0(0.0)	0.217^a^	1.000^b^
BUN increase	8(2.1)	0(0.0)	1(0.3)	0(0.0)	94(24.3)	2(0.5)	1(0.3)	0(0.0)	<0.001^a^	1.000^b^
Creatinine increase	13(3.4)	2(0.5)	0(0.0)	0(0.0)	86(22.2)	11(2.8)	2(0.5)	1(0.5)	<0.001^a^	0.499^b^

IC, induction chemotherapy; RT, radiotherapy; CCRT, concurrent chemoradiotherapy; AST, aspartate aminotransferase; ALT, alanine aminotransferase; BUN, blood urea nitrogen.

^a^P value calculated using the chi-square test. ^b^P value calculated using Fisher’s exact test.

## Discussion

In the present study, we found that IC+IMRT alone showed similar efficacy, relative to IC+CCRT, in low-risk NPC patients, but was associated with fewer acute toxicities. However, in high risk patients, IC+CCRT was superior to IC+IMRT alone. In addition, we constructed a nomogram to estimate the benefit of adding CC to IMRT after IC in individual patients.

For non-metastatic locoregionally advanced NPC, CCRT has been shown to be more effective than RT alone, and has been accepted as the standard treatment for advanced NPC (6, 7). Moreover, IC has recently received an increased amount of attention for the management of advanced NPC, and encouraging survival results have been reported for IC followed by RT alone (30, 31). Additional evidence has indicated similar survival outcomes between the IC+RT and IC+CCRT arms (15–18, 32). Our results for the whole cohort are consistent with the previous findings. Through an assessment of 217 stage II–IVB NPC patients, Liu et al. (16) reported that there was no significant difference in OS and PFS between the IC plus volumetric modulated arc therapy (VMAT) alone and IC+CCRT arms, although additional side effects were observed in the IC/CCRT arm. In their analysis of 154 patients treated with IC+RT with or without CC, Wei et al. (17) found similar results. However, only a small number of patients were enrolled in the above studies. Furthermore, in the trial conducted by Huang et al. (15), the IC+CCRT program did not yield improved OS or failure-free survival in NPC patients, relative to the IC+RT program. However, it should be noted that the platinum drug used by the researchers was carboplatin. Moreover, the study conducted by Lin et al. (32) suggested that CC offered no significant value for the further improvement of local and regional control over IMRT following IC. However, these 2 subgroups of patients were imbalanced, and <50 patients received CC among the 370 patients in this study. Moreover, the prognostic difference was only discussed in the whole cohort, and was not investigated according to the risk-stratified subgroup.

Plasma EBV DNA levels—an important prognostic factor for NPC patients—in combination with the TNM stage could help identify patients with locoregionally advanced NPC at high risk of locoregional recurrence and distant metastasis (33). In a subgroup analysis stratified by clinical stage and EBV DNA levels, we observed an interesting scenario. The subgroup analysis showed that IC+CCRT achieved better outcomes in terms of survival rate improvement and distant metastasis rate lowering in high risk patients (IVa–b or EBV DNA ≥4,000 copies/ml). However, there was no survival benefit of IC+RT alone and IC+CCRT among low risk patients (II–III with EBV DNA <4,000 copies/ml). We believe that IMRT improves the local control rate and IC decreases distant metastasis, which may “counteract” the effect of CC in improving the local control rate and survival rate in these low-risk patients who had better prognosis. Our results suggest that IC+RT alone for low-risk NPC patients can produce satisfactory results. In addition, our study found that the rates of severe Grade 3–4 hematological toxicities were lower in the IC+RT group and a lower incidence of grade 1–4 nephrotoxicity was observed in the IC+RT group. As a result, IC+RT can reduce toxicities and has comparable antitumor efficacy with IC+CCRT for low-risk NPC patients. As for high-risk patients, these patients were with high tumor burden and the intensive treatment regimen, addition of CC, could enhance the antitumor and the addition of CC is recommended.

Furthermore, we constructed a nomogram to estimate the benefit of adding CC to IMRT after IC. The nomogram would particularly help having one-to-one discussions with patients and in making treatment decisions. For example, when an NPC patient with T3 (43 points) disease, N2 (47 points) disease, and EBV DNA <4,000 copies/ml (0 points) visits the clinic, and receives IC+RT alone (31 points), s/he would accrue a total of 121 points, and would have an estimated 5-year PFS rate of 84%. If the same patient also received CC, the estimated 3-year PFS rate would be 89%, with a corresponding benefit of 5% from the CC. Considering the cost, toxicity, and side effects of CC, this patient may prefer to avoid CC, particularly due to the relatively small expected benefit. However, a patient with T4 (100 points) disease, N3 (80 points) disease, and EBV DNA ≥4,000 copies/ml (38 points) who undergoes IC+RT alone (31 points) would have a total score of 249, and a corresponding 3-year FFS of 38%. If this patient received additional CC, the total score would be 218 points, and the estimated 3-year PFS would increase to 52%, thus indicating a much greater benefit of 14% for this patient; therefore, for this patient, the addition of CC was more beneficial. Nevertheless, the administration of CC should be discussed in greater detail between the clinician and patient after multivariate evaluation before making a decision, which cannot be all included in this nomogram. Thus, the nomogram can serve as an important reference, but should not be used as the sole basis for making clinical treatment decisions for NPC patients.

The present study has several limitations. First, there was probably a selection bias due to the retrospective nature of the study. Second, data on the response to IC and the EBV DNA level after IC were not collected, which might be of great research value. Third, we have not specified a specific threshold at which CC should be recommended in this nomogram. Finally, this model has not been validated in an independent cohort. Nevertheless, we performed bootstrap validation, which provides a stringent assessment, and the model demonstrated good accuracy for predicting PFS (c-index, 0.703; **Figure 5**).

## Conclusions

The present study suggests that IC+IMRT alone showed similar efficacy, relative to IC+CCRT, in low-risk NPC patients (II–III with EBV DNA <4,000 copies/ml), but was associated with fewer acute toxicities. However, in high risk patients (IVa–b or EBV DNA ≥4,000 copies/ml), IC+CCRT was superior to IC+IMRT alone. In addition, we established a nomogram to estimate the benefit of adding CC to IMRT after IC for individual patients, which could help having one-to-one discussions with the patient and in making treatment decisions.

## Data Availability Statement

The datasets generated for this study are available on request to the corresponding authors.

## Ethics Statement

This retrospective study was approved by the Clinical Research Committee of Sun Yat-sen University Cancer Center. The patients/participants provided their written informed consent to participate in this study. Written informed consent was obtained from the individual(s) for the publication of any potentially identifiable images or data included in this article.

## Author Contributions

H-QM, LG, L-QT, and J-XB developed the study concepts. S-LL, X-SS, and Z-JL participated in the study design. S-LL, X-SS, and Z-JL participated in the data acquisition. S-LL, X-SS, and Z-JL participated in the quality control of data and algorithms. SLL and X-SS participated in the data analysis and interpretation. S-LL, X-SS, and Q-YC participated in the statistical analysis. S-LL, Q-YC, and H-XL participated in the manuscript preparation. S-LL, Q-YC, and H-XL participated in the manuscript editing. H-QM, LG, and J-XB participated in the manuscript review. All authors contributed to the article and approved the submitted version.

## Funding

This work was supported by grants from the National Key R&D Program of China (2017YFC0908500, 2017YFC1309003), the National Natural Science Foundation of China (No. 81425018, No. 81672868, No.81802775), the Sci-Tech Project Foundation of Guangzhou City (201707020039), the Sun Yat-sen University Clinical Research 5010 Program, the Special Support Plan of Guangdong Province (No. 2014TX01R145), the Natural Science Foundation of Guangdong Province (No.2017A030312003, No.2018A0303131004), the Natural Science Foundation of Guangdong Province for Distinguished Young Scholar (No. 2018B030306001), the Sci-Tech Project Foundation of Guangdong Province (No. 2014A020212103), the Health & Medical Collaborative Innovation Project of Guangzhou City (No. 201400000001, No.201803040003), Pearl River S&T Nova Program of Guangzhou (No. 201806010135), the Planned Science and Technology Project of Guangdong Province (2019B020230002), the National Science & Technology Pillar Program during the Twelfth Five-Year Plan Period (No. 2014BAI09B10), Natural Science Foundation of Guangdong Province (2017A030312003, and the Fundamental Research Funds for the Central Universities.

## Conflict of Interest

The authors declare that the research was conducted in the absence of any commercial or financial relationships that could be construed as a potential conflict of interest.

## Supplementary Material

Supplementary Figure 1 Kaplan–Meier progression-free survival curves in 1,324 NPC patients. **(A)** Patients are grouped based on the EBV DNA levels (≥4,000 vs. <4,000 copies/ml); **(B)** overall stage (IVa–b vs. II–III); **(C)** combination of EBV DNA (≥4,000 vs. <4,000 copies/ml) and overall stage (IVa–b vs. II–III); and **(D)** risk stratification.

## Abbreviations

NPC, nasopharyngeal carcinoma; RT, radiotherapy; CC, concurrent chemotherapy; AC, adjuvant chemotherapy; IC, induction chemotherapy; CCRT, concurrent chemoradiotherapy; IMRT, intensity-modulated radiotherapy; OS, overall survival; PFS, progression-free survival; LRFS, locoregional relapse-free survival; DMFS, distant metastasis-free survival; WHO, World Health Organization; EBV, Epstein–Barr virus; TPF, taxanes, cisplatin and 5-fluorouracil; PF, cisplatin and 5-fluorouracil; TP, taxanes and cisplatin; MRI, magnetic resonance imaging; CT, computed tomography; PET/CT, positron emission tomography/computed tomography; PSM, propensity score matching;HR, hazard ratio; CIs, confidence intervals.
