# Supplementary material for: Nomogram Predicting the Benefits of Adding Concurrent Chemotherapy to Intensity-Modulated Radiotherapy After Induction Chemotherapy in Stages II–IVb Nasopharyngeal Carcinoma
Source: Front Oncol. 2020 Nov 9;10:539321. doi: 10.3389/fonc.2020.539321 (PMC7681000; doi:10.3389/fonc.2020.539321)
Supplement: Supplementary file 1 [file Table_1.docx]

Supplement Table 1. Summary of multivariate analyses of prognostic factors for 3-year progression-free survival in 295 high-risk NPC cases.

|  | **Hazard ratio* (95% CI)** | **P value** |
| --- | --- | --- |
| **Progression-free survival** |  |  |
| Age (y) (≥ 45 vs. < 45) | 1.570(0.927-2.660) | 0.094 |
| Gender(F vs. M) | 1.101(0.624-1.943) | 0.740 |
| T category;T3 vs.T1-2 | 1.451(0.552-3.810) | 0. 450 |
| T category;T4 vs.T1-2 | 2.251(0.811-6.249) | 0.119 |
| N category; N2 vs. N0-1 | 1.435(0.783-2.631) | 0.243 |
| N category; N3 vs. N0-1 | 1.972(0.951-4.092) | 0.243 |
| EBV DNA | 1.143(0.601-2.176) | 0.683 |
| Regimen; IC+CCRT vs. IC+RT | 0.422(0.252-0.706) | 0.001 |
| **Distant metastasis-free survival** |  |  |
| Age (y) (≥ 45 vs. < 45) | 2.512(1.243-5.076) | 0.010 |
| Gender(M vs. F) | 0.874(0.428-1.785) | 0.973 |
| T category;T3 vs.T1-2 | 1.019(0.338-3.072) | 0.973 |
| T category;T4 vs.T1-2 | 1.160(0.348-3.871) | 0.809 |
| N category; N2 vs. N0-1 | 1.390(0.620-3.115) | 0.424 |
| N category; N3 vs. N0-1 | 1.992(0.778-5.096) | 0.151 |
| EBV DNA | 1.332(0.554-3.201) | 0.522 |
| Regimen; IC+CCRT vs. IC+RT | 0.347(0.175-0.688) | 0.002 |

CI, confidence interval; EBV, Epstein–Barr virus; IC, induction chemotherapy；RT, radiotherapy; CCRT, concurrent chemoradiotherapy.

A Cox proportional hazards regression model was used to detect variables, one-by-one, without adjustment. All variables were transformed into categorical variables. HRs were calculated for age (≥45 *vs*. <45 years); sex (female *vs*. male); T stage (3 *vs*. 1–2; 4 *vs*. 1–2); N stage (2 *vs*. 0–1; 3 *vs*. 0–1); EBV DNA level (≥4000 copies/ml *vs*. <4000 copies/ml); and regimen (IC+CCRT *vs*. IC+RT).
