# Supplementary figures and images for: Nomogram Predicting the Benefits of Adding Concurrent Chemotherapy to Intensity-Modulated Radiotherapy After Induction Chemotherapy in Stages II–IVb Nasopharyngeal Carcinoma
Source: Front Oncol. 2020 Nov 9;10:539321. doi: 10.3389/fonc.2020.539321 (PMC7681000; doi:10.3389/fonc.2020.539321)

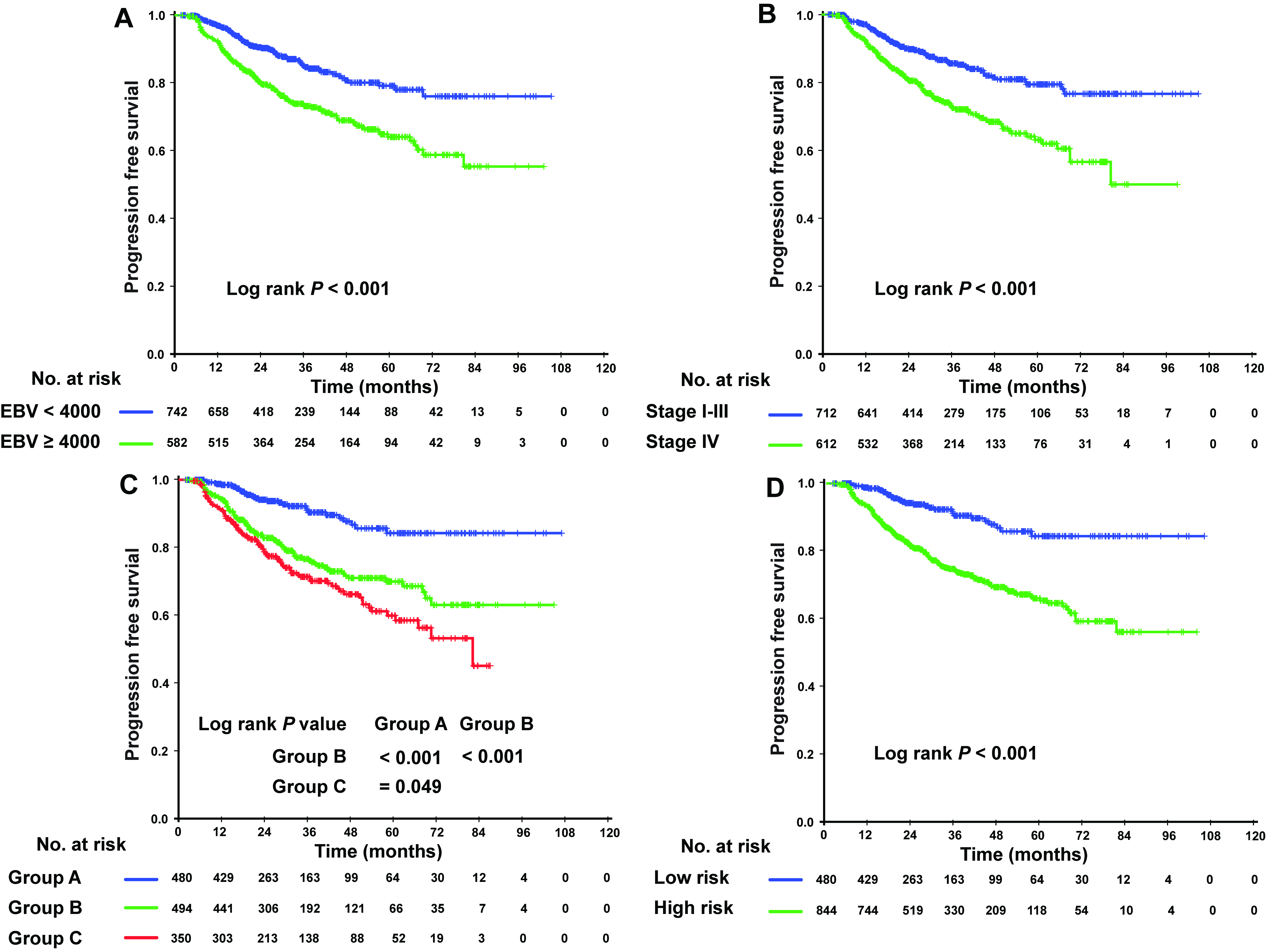

Supplement: Supplementary Figure 1 — Kaplan–Meier progression-free survival curves in 1,324 NPC patients. (A) Patients are grouped based on the EBV DNA levels (≥4,000 vs. <4,000 copies/ml); (B) overall stage (IVa–b vs. II–III); (C) combination of EBV DNA (≥4,000 vs. <4,000 copies/ml) and overall stage (IVa–b vs. II–III); and (D) risk stratification. [file Image_1.tif]
